# Comparative evaluation of machine learning strategies for short-term dengue forecasting in Brazilian capital municipalities

**DOI:** 10.1007/s00484-026-03300-7

**Published:** 2026-08-14

**Authors:** Darllan Collins da Cunha e Silva, Liliane Moreira Nery, Nícholas de Paula Nicomedes, Pedro Cesar Madureira de Godoy Camargo, Leopoldo André Dutra Lusquino Filho, Raphael de Vicq Ferreira da Costa, Teresa Maria Fernandes Valente

**Affiliations:** 1 São Paulo State University (UNESP), Institute of Science and Technology, Sorocaba, Brazil; 2 University of Minho, Institute of Earth Sciences (ICT), Braga, Portugal

**Keywords:** Epidemiological surveillance, Time series, Multi-horizon forecasting, Public health, Predictive modeling

## Abstract

Dengue is an arboviral disease of high public health relevance, characterized by pronounced temporal variability, nonlinearity, and recurrent outbreaks, which pose challenges to epidemiological surveillance and decision-making. This study evaluated the performance of machine learning methods for short-term forecasting of the weekly dengue morbidity rate in the 27 Brazilian capital cities, comprising the 26 state capitals and Brasília, Federal District, with horizons up to 4 weeks. Epidemiological, climatic, and socioeconomic data were compiled for these capital cities and used to compare a Gated Recurrent Unit (GRU) neural network, formulated as a Multi-Input Multi-Output (MIMO) model, and a Gradient Boosting model (CatBoost), implemented using a Direct forecasting strategy with horizon-specific models. Validation was conducted using a walk-forward approach, with evaluation based on absolute error metrics and the coefficient of determination. The results indicated that the GRU architecture presented recurring limitations, including underfitting, temporal lag, and low capacity to anticipate epidemic peaks. In contrast, the CatBoost model demonstrated greater robustness and better adaptation to the variability of epidemiological time series, showing superior performance in most of the analyzed capitals. The findings reinforce that greater architectural complexity does not necessarily imply better operational performance and highlight the potential of ensemble-based methods for short-term epidemiological surveillance applications. These findings contribute to dengue forecasting by showing that, under a common validation framework, ensemble-based strategies may provide greater operational robustness than recurrent MIMO architectures for short-term prediction in heterogeneous epidemiological settings.

**Supplementary Information:**

The online version contains supplementary material available at 10.1007/s00484-026-03300-7.

## Introduction

Dengue is an arboviral disease of global relevance, characterized by a consistent increase in incidence, geographic expansion, and recurrence of outbreaks, especially in tropical and subtropical regions where environmental and socio-spatial factors favor transmission (Johansson et al. 2019). The recent increase in case numbers, observed across the Region of the Americas, reinforces the need for more effective analytical and predictive approaches to support surveillance and public health response (WHO 2024, 2025; Sebastianelli et al. 2024).

In Brazil, the high magnitude of dengue results from the combination of a tropical climate, intense urbanization, and socioeconomic heterogeneity, which favor the persistence of Aedes aegypti and the recurrence of outbreaks (Simon and Rangel 2021; Cordeiro et al. 2020; Andrade et al. 2024). The country consistently ranks among those with the highest disease burden in the Americas, with recent records exceeding historical levels and indicating increasing pressure on surveillance systems and public health services (PAHO and WHO 2024; Brasil 2024), establishing a strategic context for spatiotemporal studies and the development of predictive models at the national scale.

Dengue transmission is conditioned by the interactions among climatic variables, urbanization, and socioeconomic factors, which operate nonlinearly and with temporal lags. Temperature influences the life cycle of Aedes aegypti, while precipitation regulates the availability of breeding sites, particularly in urban environments with limited infrastructure (Viana and Ignotti 2013; Duarte et al. 2019). Additionally, population density, social inequality, and access to sanitation modulate human exposure and local vulnerability, contributing to the recurrence of outbreaks and to heterogeneous endemic patterns across space and time (Kikuti et al. 2015; Zellweger et al. 2017; Sousa et al. 2021). This territorial heterogeneity is consistent with broader evidence from Brazilian spatial epidemiology showing that socioeconomic and environmental indicators shape health vulnerability patterns across regions (Nicomedes et al. 2026).

Short-term forecasting is essential for dengue surveillance, as it enables anticipating higher-risk periods and supporting operational public health actions (Lowe et al. 2014; Souza et al. 2022). Weekly forecasts are particularly relevant because they capture the seasonality, variability, and epidemic peaks characteristic of the disease, although they face challenges related to data nonlinearity and spatial heterogeneity (Johansson et al. 2019; Codeco et al. 2018; Mussumeci and Coelho 2020).

Machine learning methods have been widely applied in dengue forecasting due to their ability to capture nonlinear relationships in complex epidemiological time series (Guo et al. 2017). In Brazil, studies indicate that predictive gains depend on model architecture, forecasting horizon, and spatial scale, with recurring limitations in regional generalization and in anticipating epidemic peaks (Souza et al. 2022; Mussumeci and Coelho 2020; Johansson et al. 2019; Nery et al. 2026). Ensemble-based and nowcasting approaches have shown greater robustness for operational applications when integrated with climatic and epidemiological data (Codeco et al. 2018; Sebastianelli et al. 2024; Chen and Moraga 2025).

This study evaluates the performance of machine learning methods for short-term forecasting of the weekly dengue morbidity rate in the 27 Brazilian capital cities, comprising the 26 state capitals and Brasília, Federal District, with horizons up to 4 weeks. To this end, the performance of a MIMO GRU architecture was compared with a CatBoost model using a Direct strategy, allowing the identification of limitations of recurrent models in anticipating epidemic peaks and the greater robustness of ensemble-based approaches for irregular epidemiological time series in contexts of high climatic and socioeconomic heterogeneity, thereby contributing to the improvement of predictive applications aimed at epidemiological surveillance and public health decision-making.

The contribution of this study is methodological and applied, focusing on the operational performance of machine learning strategies for dengue forecasting. By comparing GRU-MIMO and CatBoost-Direct models using the same capital-city dataset, forecast horizons, and walk-forward validation framework, the study assesses whether greater architectural complexity translates into more reliable short-term predictions.

## Materials and methods

### Study area

**Fig. 1:**
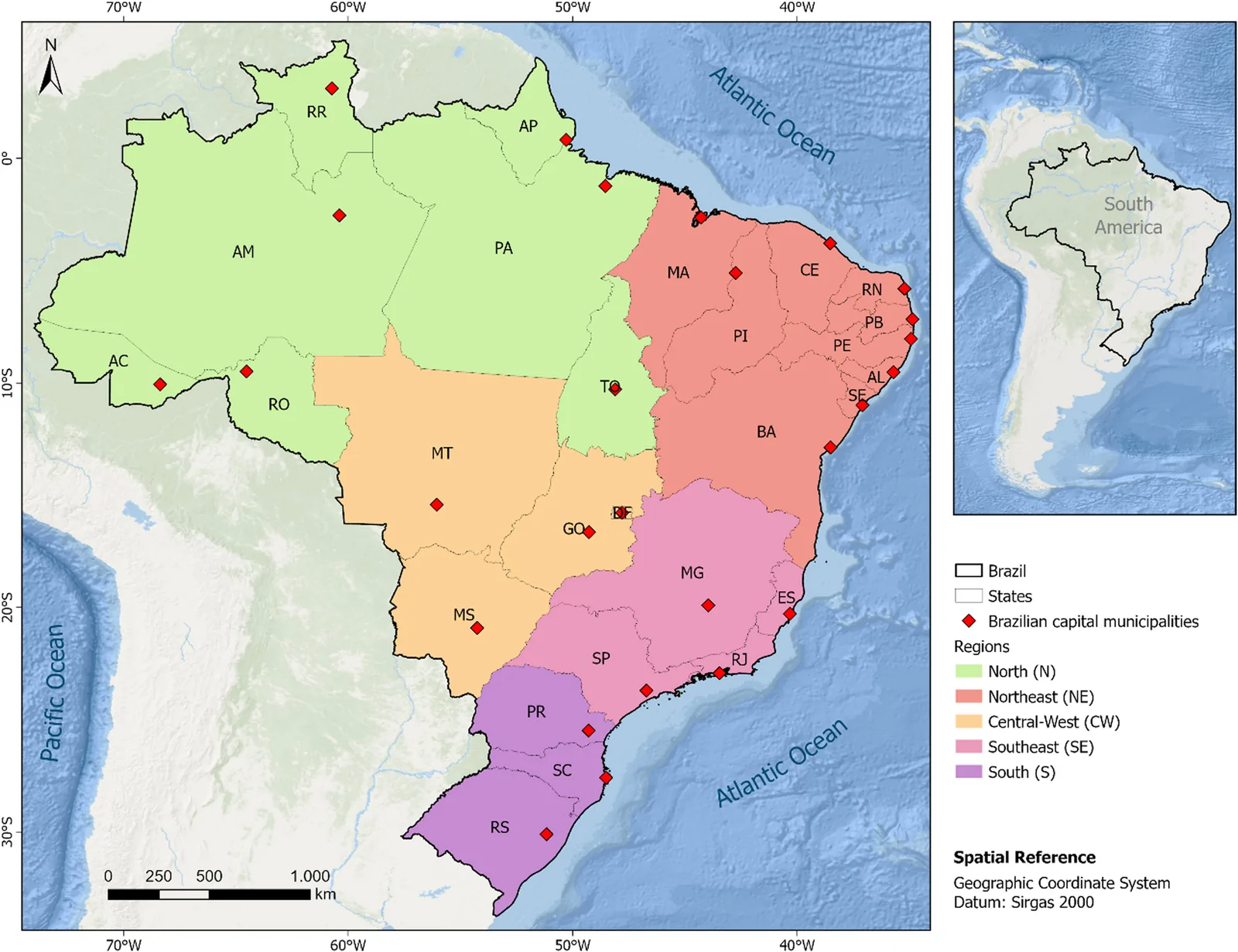
Spatial distribution of the 27 Brazilian capital municipalities analyzed in this study

The study was conducted in the 27 Brazilian capital municipalities, comprising the 26 state capitals and Brasília, Federal District (Fig. 1). The capital municipalities are distributed across the five Brazilian geographic regions and represent heterogeneous climatic, demographic, and socioeconomic contexts relevant to dengue transmission. Brazil, which provides the national geographic context of the analysis, is the largest country in South America, with a territorial extent of approximately 8,510,000 km² and approximately 203,080,756 inhabitants (IBGE 2022). It has an annual Gross Domestic Product (GDP) of R$ 10.9 trillion (2023) and a GDP per capita of R$ 42,247.52 (IBGE 2022).

Brazil is predominantly located in the tropical zone and is characterized by high temperatures and high levels of precipitation. Due to its location in low-latitude regions, Brazil is classified as a tropical country, with hot and humid climates predominating and average temperatures around 20 °C (Viana and Ignotti 2013). These climatic conditions are favorable for the proliferation of the mosquito Aedes aegypti, the vector responsible for dengue transmission. Due to the country’s tropical characteristics, Brazil has experienced several epidemics, particularly in 2013, when approximately 2 million cases occurred, driven by conditions that favor the proliferation of the main disease vector (Sobral and Sobral 2019).

According to the PAHO and WHO (2024), in the Region of the Americas, between epidemiological weeks (EW) 1 and 26 of 2024, a total of 10,576,561 suspected dengue cases were reported, with 5,618 deaths recorded. In Brazil, in addition to the impacts of meteorological anomalies, the vast territorial extent, large population size, and the high sensitivity and capacity of the surveillance system contribute to the country standing out when data are compared in absolute numbers (Brasil 2024). Therefore, the combination of the tropical climate with socio-environmental factors makes dengue a constant public health concern in Brazil, requiring ongoing prevention and control measures.

### Data acquisition and processing

Annual data were collected for the 27 Brazilian capital municipalities between 1999 and 2021, referring to dengue morbidity from hospitalizations reported in the Brazilian Unified Health System (DATASUS), classified according to ICD-10, including typologies A90 and A91, corresponding to dengue and dengue hemorrhagic fever, respectively.

In addition, data on GDP per capita and municipal population were obtained from the Brazilian Institute of Geography and Statistics (IBGE), while Purchasing Power Parity (PPP) data were obtained from the World Bank, and the extent of urbanized areas at the municipal level was obtained from MapBiomas.

The data were subjected to a preprocessing procedure to ensure consistency and compatibility across sources, using the municipality code as the unique identifier. This procedure enabled the construction of a Brazilian capital municipal-level database.

### Dengue morbidity rate and socioeconomic indicators

Morbidity data were analyzed using a rate per 100,000 inhabitants (Eq. 1), with the municipal population of each analyzed year as the basis.

1 $$\:{Tx}_{Dengue}=\:\frac{{Morb}_{municipality}}{{Pop}_{municipality}}\:\times\:\mathrm{100,000}$$

Where:

$\:{Tx}_{Dengue}$ :is the dengue morbidity rate for each Brazilian capital municipality;

$\:{Morb}_{municipality}$ :corresponds to the raw number of morbidities from hospitalization cases of dengue occurring during a one-year period for each Brazilian capital municipality;

$\:{Pop}_{municipality}$ :corresponds to the raw population number of each Brazilian capital municipality reported by IBGE.

#### Descriptive characterization of dengue morbidity data

Before model training, the dengue morbidity series were annually descriptively analyzed for the 27 Brazilian capital municipalities (Supplementary Table 1). The series showed a sparse and strongly right-skewed distribution, with annual zero records ranging from 10% to 59%. In all years, the mean was higher than the median, and the standard deviation exceeded the mean, indicating high dispersion and the influence of epidemic peaks. This pattern was also reflected in the high coefficients of variation, which ranged from 162% to 310%, and in the contrast between low median values and high annual maxima, reaching 60.5526 cases per 100,000 inhabitants. These characteristics indicate an irregular, sparse, and peak-driven temporal structure, as shown in Supplementary Table 1. This pattern supported the use of the log1p transformation and robust loss functions to reduce the influence of asymmetry, zeros, and extreme values (Kurz 2017; Kokonendji et al. 2021; Xu and Lou 2023), as well as autoregressive lags, moving-window statistics, and walk-forward validation to account for temporal dependence in dengue forecasting (Johansson et al. 2019; Mussumeci and Coelho 2020).

### Socioeconomic and environmental indicators

On the other hand, for the calculation of population density, population data were divided by the area of each municipality, based on a vector file containing municipal boundaries provided by IBGE.

In the case of the proposed population financial power index, it was based on the relationship between municipal GDP per capita and the PPP indicator, which is essential for international economic comparisons by adjusting exchange rates to reflect price levels across countries. Thus, PPP indicates the relative price of a specific basket of goods and services in each of the compared economies, taking a reference economy as a baseline (Rissanen and Inklaar 2023), allowing a more accurate assessment of economic well-being and global development.

GDP per capita data were collected for the 27 Brazilian capital municipalities from IBGE, and PPP data for extreme poverty (Table 1) were obtained from the World Bank between 1999 and 2021 and applied in Eq. 2. Since PPP is expressed in US dollars (US$) per day, this value was adjusted to Brazilian Real (BRL) per year. For this adjustment, the US dollar (US$) exchange rate for each analyzed year, provided by IPEA (Institute for Applied Economic Research), served as the basis. The equation (Eq. 2) used is described as follows:

2 $$\:{I}_{PFP}=\:\frac{{GDP\:per\:capita}_{Municipal}}{{PPP}_{annual}}$$

Where:

$\:{I}_{PFP}$ :Population Financial Power Index;

$\:{GDP\:per\:capita}_{Municipal}$ :is the value of municipal GDP per capita for each year of the studied period;

$\:{PPP}_{annual}$ :corresponds to the purchasing power parity value provided by the World Bank for each year during the studied period (Table 1).

**Table 1:** PPP between 1999 and 2021

Year	Extreme poverty (PPP)
1999–2004	US$ 1.08/day
2005–2010	US$ 1.25/ day
2011–2021	US$ 1.90/ day
2022 – Present	US$ 2.15/ day

Source: World Bank

Finally, for the urbanization index, the ratio of urbanized areas to the total municipal area was calculated from Land Use and Land Cover (LULC) maps provided by the MapBiomas project (https://brasil.mapbiomas.org/) for each year.

### Climatic variables

Climatic conditions may influence the spread of vectors, such as dengue. Thus, climatic data were collected, including annual precipitation, minimum relative humidity, and maximum temperature recorded for each year of the study period for each Brazilian municipality. The data used in this research are from the Brazilian gridded daily meteorological dataset developed by Xavier et al. (2022) and made available on the online platform (https://sites.google.com/site/alexandrecandidoxavierufes/home). The available files are in GeoTIFF format, with a spatial resolution of 0.1° × 0.1°, which were reprojected to the Albers equal-area projection, as recommended by IBGE. Subsequently, the mean values of the studied variables were obtained for each municipality using the Zonal Statistics tool available in ArcGIS.

### Recurrent artificial neural network

To forecast dengue morbidity rates a GRU-based recurrent neural network was implemented.

#### Preprocessing

Based on the assumption and objective of developing a short-term forecasting model (horizon H = 4 weeks) for the weekly dengue morbidity rate at the municipal level, the target variable ($\:{y}_{t}$) was preprocessed using the log1p transformation to stabilize the scale and reduce data asymmetry. The exogenous covariates were normalized using a Min-Max scaler, and a window-based scaler was employed during the walk-forward validation process to prevent leakage of future temporal data.

3 $$\:{\stackrel{\sim}{y}}_{t}=\:log\:(1+{y}_{t})$$

4 $$\:{y}_{t}=exp\:\left({\stackrel{\sim}{y}}_{t}\right)\:-\:1$$

#### Feature engineering

Classical autoregressive lags were used $\:({y}_{t}-1,\:{y}_{t}-2,{y}_{t}-4,\:{y}_{t}-52y)$, along with moving statistics (mean, maximum, standard deviation) calculated over windows of 4, 8, and 12 weeks, and growth rate indicators ($\:\left[{\varDelta\:y}_{t}\right]$, percentage variation, and log-difference). To explicitly model interannual cycles (52 weeks), periodic encodings (Eqs. 5 and 6) were used for the week of the year (52-cycle) and for the month (12-cycle), as well as a simple additive decomposition (Eq. 7).

5 $$\:sin\:\left(\frac{2\pi\:\:week}{52}\right),\:cos\:\left(\frac{2\pi\:\:week}{52}\right)$$

6 $$\:sin\:\left(\frac{2\pi\:\:month}{12}\right),\:cos\:\left(\frac{2\pi\:\:month}{12}\right)$$

7 $$\:{y}_{t}\:=\:{trend}_{t}\:+\:{seasonal}_{t}\:+\:{residual}_{t}$$

Where:

$\:{trend}_{t}$ :is the approximate trend through a 52-period centered moving average; 

$\:\:{seasonal}_{t}$: is the average pattern over the cycles; 

$\:\:{residual}_{t}$:is the unexplained component.

#### Model architecture

The forecasting task was formulated as a MIMO problem, in which the model analyzes multiple past inputs simultaneously (Multi-Input) to predict morbidity rates for multiple future weeks (Multi-Output) (Taieb et al. 2009, 2010). Thus, a single encoder was used: a Recurrent Artificial Neural Network (GRU) (Eq. 8), due to its performance and computational efficiency (Cho et al. 2014). In addition, horizon-specific decoders were employed for each predicted week (Eq. 9), conditioned on a horizon embedding (eₕ ∈ ℝ^(dₑ), making the model sensitive to differences across forecasting steps and improving accuracy at longer horizons without increasing the number of models (Lim et al. 2021).

8 $$\:{h}_{l}=GRU\left({X}_{1},\dots\:,{X}_{L}\right)\in\:{R}^{{d}_{h}}$$

9 $$\:\widehat{{y}_{t+h}}={w}_{h}^{\top\:}\left[{h}_{l};{e}_{h}\right]+{b}_{h}$$

Where:

$\:\widehat{{y}_{t+h}}$ :is the model prediction for a specific week, where * h* represents the forecast horizon;

$\:{w}_{h}^{\top\:}$ and $\:{b}_{h}$: are the weights (* w*) and bias (* b*) used during training to connect the past summary ($\:{h}_{l})$ to the final prediction ($\:\widehat{{y}_{t+h}}$);

$\:{e}_{h}$ :is the horizon embedding, which informs the model about the forecast horizon (* h*).

The model was trained and evaluated under three distinct hyperparameter configurations to assess the trade-off between model capacity, the size of the historical window (in weeks), and the robustness of the loss function. All configurations share the MIMO architecture with horizon embeddings (dimension 8) and horizon-specific decoders.

Configuration 1 was designed for maximum capacity, using a long historical window (104 weeks), greater network depth, and the Tweedie loss function (*p* = 1.5), which is methodologically appropriate for semi-continuous data with zeros (Kurz 2017; Kokonendji et al. 2021). Configurations 2 and 3 used the MAE (Mean Absolute Error) loss function, known for its robustness to outliers (Xu and Lou 2023), while testing, respectively, an intermediate network capacity (Configuration 2) and a shorter historical window with lower capacity (Configuration 3).

The input vector at each timestep has an exact dimension of 18 + N, where N represents the number of neighboring municipalities. This vector comprises the current value of the target, N neighborhood columns, 12 columns of lagged climatic variables (lags 1, 2, 4, and 8 for 3 variables), 3 static variables, and 2 calendar components. The exogenous covariates are incorporated into the model by concatenating them along the feature axis at each temporal step. To handle different forecast horizons (h = [1, 4]), a horizon embedding was used, integrated into the network’s hidden state via concatenation before the dense output layer. The input windows (lookback) of 52 and 104 weeks were selected empirically, based on the duration of the interannual cycle and on previous experiments that showed shorter or excessively long windows led to a loss of performance in capturing the annual and biennial seasonality of dengue.

The details of each configuration are summarized in Table 2. Training in all configurations used early stopping to prevent overfitting (excessive adjustment to training data) and learning rate optimization based on plateau.

**Table 2:** Summary of the hyperparameter configurations evaluated for the GRU model

Hyperparameter	Configuration 1	Configuration 2	Configuration 3
Horizon	104 weeks	104 weeks	52 weeks
GRU Layers	3	2	2
Neurons / Hidden Layer	256	128	64
Dropout	0.3	0,2	0.1
Loss Function	Tweedie (*p* = 1.5)	MAE	MAE
Batch Size	32	32	64
Learning Rate	0.00050	0.0010	0.0010
Training Epochs	15 epochs	15 epochs	12 epochs

### Estimation of morbidity cases using gradient boosting

For comparative purposes, a CatBoost-based Gradient Boosting approach was applied to the 27 Brazilian state capitals.

#### Preprocessing and validation

For the forecasting of the morbidity rate, the target variable was similarly preprocessed using the $\:log\:(1+{y}_{t})$ transformation. During the prediction stage, the scale was reverted using the exponential function to return the variable to its original scale. Model validation was performed using a walk-forward approach with an expanding training window, divided into 13 folds (validation windows), where each fold generated forecasts for the subsequent 4 weeks.

#### Feature engineering

A set of lags, moving statistics (such as means and standard deviations over different windows), and differences of the target series itself was applied. Additionally, neighborhood variables (aggregated data from nearby municipalities), climatic data (with their respective lags), components of seasonal decomposition, and calendar attributes (week of the year and month) were incorporated.

#### Model architecture

For this approach, the CatBoost Regressor model, an implementation of gradient boosting decision trees (Prokhorenkova et al. 2018; Ibrahim et al. 2020), was used. Unlike the MIMO architecture used in the GRU, a Direct forecasting strategy was adopted, in which independent models were trained for each forecast horizon. Thus, four independent models were trained, with one specialist model for each forecast horizon (H1, H2, H3, and H4). All models were trained with a standardized hyperparameter configuration (including depth, learning rate, and regularization) and used Mean Absolute Error (MAE) as the loss function.

Unlike the sequential approach of the GRU, CatBoost uses a tabular data structure, treating temporal lags as independent variables, yielding an input vector of 27 + N dimensions. The hyperparameters optimized via grid search and experimentally validated were: depth 6, learning rate 0.05, and a limit of 550 trees, with an early stopping criterion of 50 rounds to prevent overfitting. No additional regularization was applied beyond the algorithm’s native defaults. It is important to note that both models (GRU and CatBoost) used the same dataset and variables (climate, calendar, and neighborhood), differing only in their processing approach (Sequential vs. Tabular).

### Evaluation metrics

To objectively evaluate and compare the predictive performance of the GRU and CatBoost models, a standardized set of error metrics was employed (Table 3). All metrics were calculated on the original data scale (morbidity rate), after reversing the logarithmic transformation applied during preprocessing.

**Table 3:** Evaluation metrics of the performance of the machine learning models used

Metric	Equation	Index
Mean Absolute Error	$\:MAE=\frac1n\sum_{i=1}^n\left|y_i-\widehat{y_i}\right|$	(10)
Root Mean Squared Error	$\:RMSE=\sqrt{\frac1n\sum_{i=1}^n\left(y_i-\widehat{y_i}\right)^2}$	(11)
Coefficient of Determination	$\:R^2=1-\frac{{\displaystyle\sum_{i=1}^n}\left(y_i-\widehat{y_i}\right)^2}{{\displaystyle\sum_{i=1}^n}\left(y_i-\overset-y\right)^2}$	(12)
Symmetric Mean Absolute Percentage Error	$\:sMAPE\left(\%\right)=\frac{100}n\sum_{i=1}^n\frac{2\left|y_i-\widehat{y_i}\right|}{\left|y_i\right|+\left|\widehat{y_i}\right|+\epsilon}$	(13)

## Results and discussion

The results are interpreted as an applied assessment of model suitability for operational dengue forecasting, emphasizing predictive robustness, temporal alignment, and sensitivity to epidemic peaks. As shown in Table 4, although Configuration 1 used the Tweedie loss function to minimize the effects of frequent zero-rate inputs, this GRU architecture was the least selected as the best configuration, based on the analyzed performance metrics.

**Table 4:** Performance results of the GRU configurations used

Capital municipality	Best GRU configuration	MAE	RMSE	*R*²	sMAPE
Aracaju	Configuration 1	0.1889	0.2678	0.5098	74%
Belém	Configuration 2	0.0161	0.0202	0.1134	61%
Belo Horizonte	Configuration 2	0.0328	0.0554	-0.2386	64%
Boa Vista	Configuration 2	0.2061	0.3291	0.014	100%
Brasília	Configuration 3	0.416	0.576	0.4761	61%
Campo Grande	Configuration 2	0.2449	0.3442	0.1175	68%
Cuiabá	Configuration 2	0.0422	0.0544	-0.404	70%
Curitiba	Configuration 2	0.0094	0.0176	-0.3822	99%
Florianópolis	Configuration 3	0.3073	0.5729	-0.0619	129%
Fortaleza	Configuration 3	0.2622	0.315	-0.5299	88%
Goiânia	Configuration 3	1.4575	2.1474	-0.0285	103%
João Pessoa	Configuration 2	0.3181	0.3973	-0.1748	100%
Macapá	Configuration 1	0.0374	0.0453	-0.3348	74%
Maceió	Configuration 3	0.1769	0.2357	0.1563	87%
Manaus	Configuration 2	0.0985	0.1286	-0.8235	75%
Natal	Configuration 3	0.2446	0.3564	0.3694	92%
Palmas	Configuration 2	0.4856	0.7561	-0.2691	110%
Porto Alegre	Configuration 2	0.2315	0.4764	-0.3085	97%
Porto Velho	Configuration 1	0.6115	0.8473	-1.0419	107%
Recife	Configuration 1	0.041	0.05	-0.8132	48%
Rio Branco	Configuration 1	0.0873	0.1245	-0.3102	65%
Rio de Janeiro	Configuration 1	0.0116	0.0164	-0.3478	75%
Salvador	Configuration 3	0.0459	0.0571	0.0463	85%
São Luís	Configuration 3	0.2095	0.3108	-0.4427	97%
São Paulo	Configuration 3	0.0141	0.0228	0.5038	111%
Teresina	Configuration 3	0.8347	1.2145	0.2308	98%
Vitória	Configuration 2	0.0479	0.0910	-0.2672	162%

This is the most complex architecture used, with 3 GRU hidden layers, each with 256 neurons. Configurations 2 and 3 were developed with 2 hidden layers, with 128 and 64 neurons, respectively. The fact that the simpler models (2 and 3) performed better in most of the studied capitals suggests that the more complex model (Configuration 1) suffered from overfitting or had difficulty generalizing to forecast during the walk-forward procedure.

It is also noteworthy that Configurations 2 and 3 used MAE, a simpler loss function, suggesting that MAE’s robustness was more beneficial for training and outlier identification. Furthermore, Configuration 3, which was the best in 10 capitals (as shown in Table 4), used only 52 weeks (1 year) of historical data, while Configurations 1 and 2 used 104 weeks (2 years). Therefore, it can be inferred that more recent historical data may be a more relevant predictor, minimizing the effects of outdated trends in the model.

However, even considering the results presented in Table 4, the GRU model failed to capture the dynamics of dengue morbidity in most capitals, as indicated by the negative R² values. Although Rio de Janeiro, Curitiba, and Belo Horizonte presented the lowest MAE and RMSE values, the use of these models is not viable due to the negative R² values, demonstrating the model’s inability to capture any epidemic variation.

**Fig. 2:**
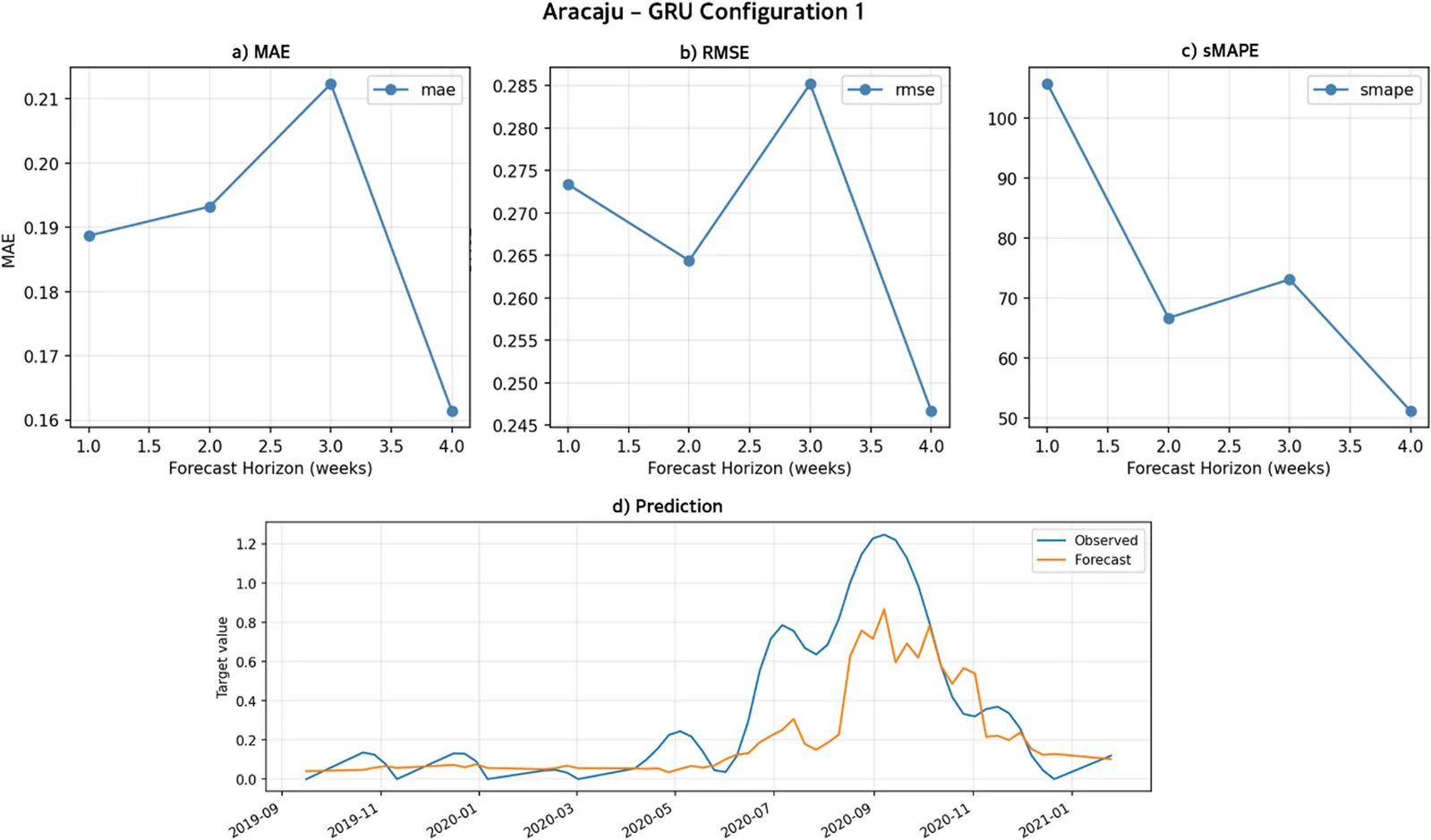
Performance of the GRU model for the capital municipality of Aracaju

**Fig. 3:**
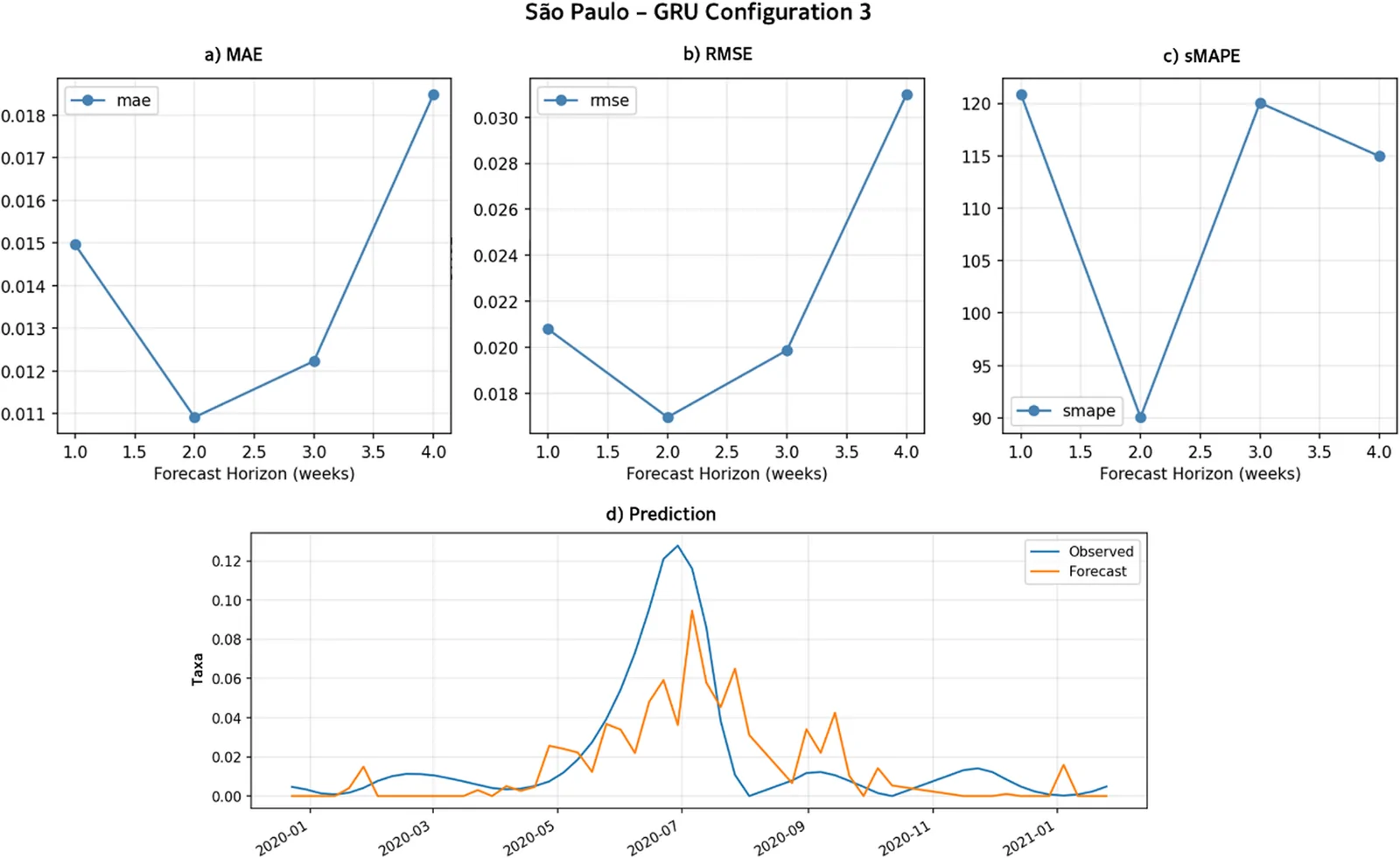
Performance of the GRU model for the capital municipality of São Paulo

**Fig. 4:**
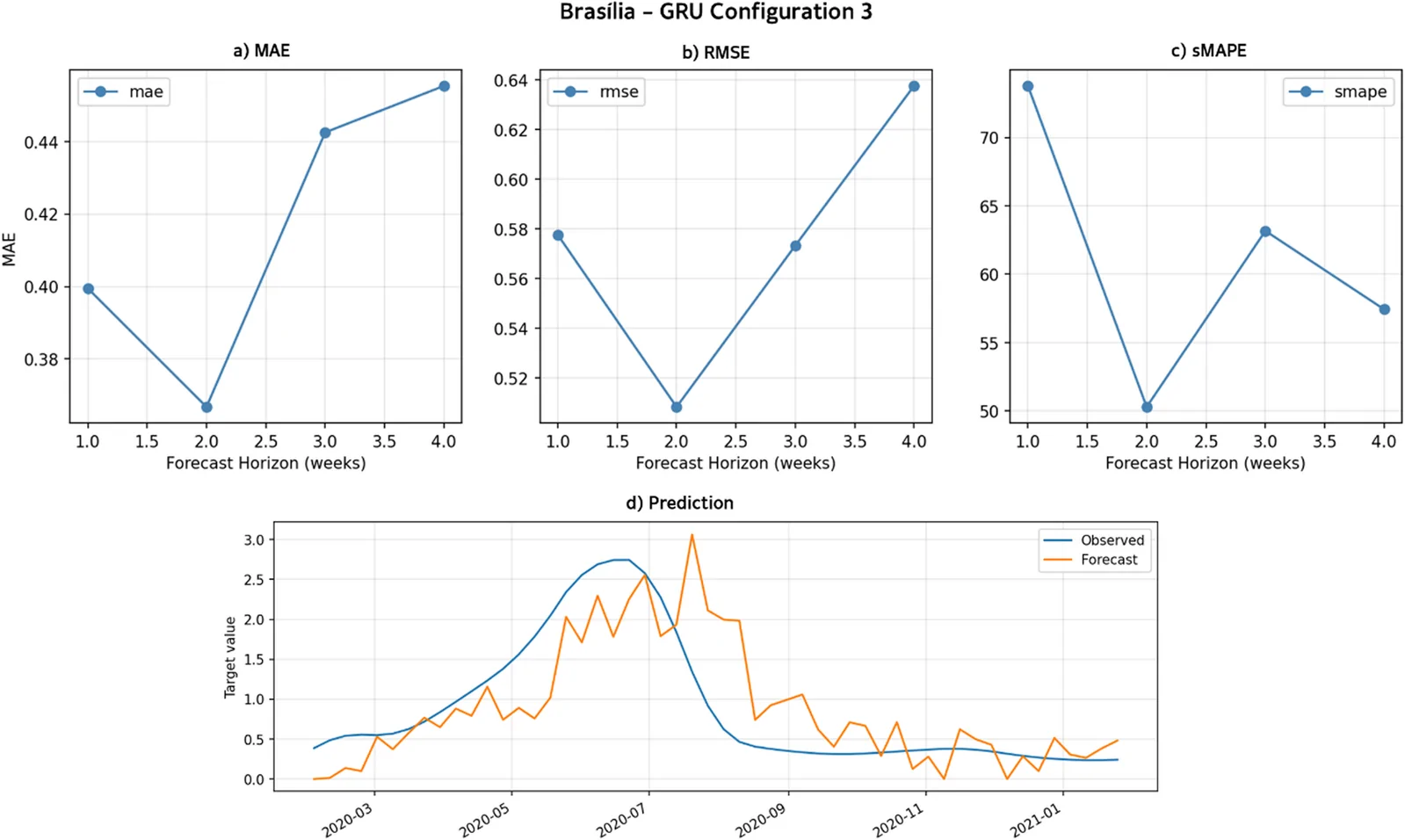
Performance of the GRU model for the capital municipality of Brasília

**Fig. 5:**
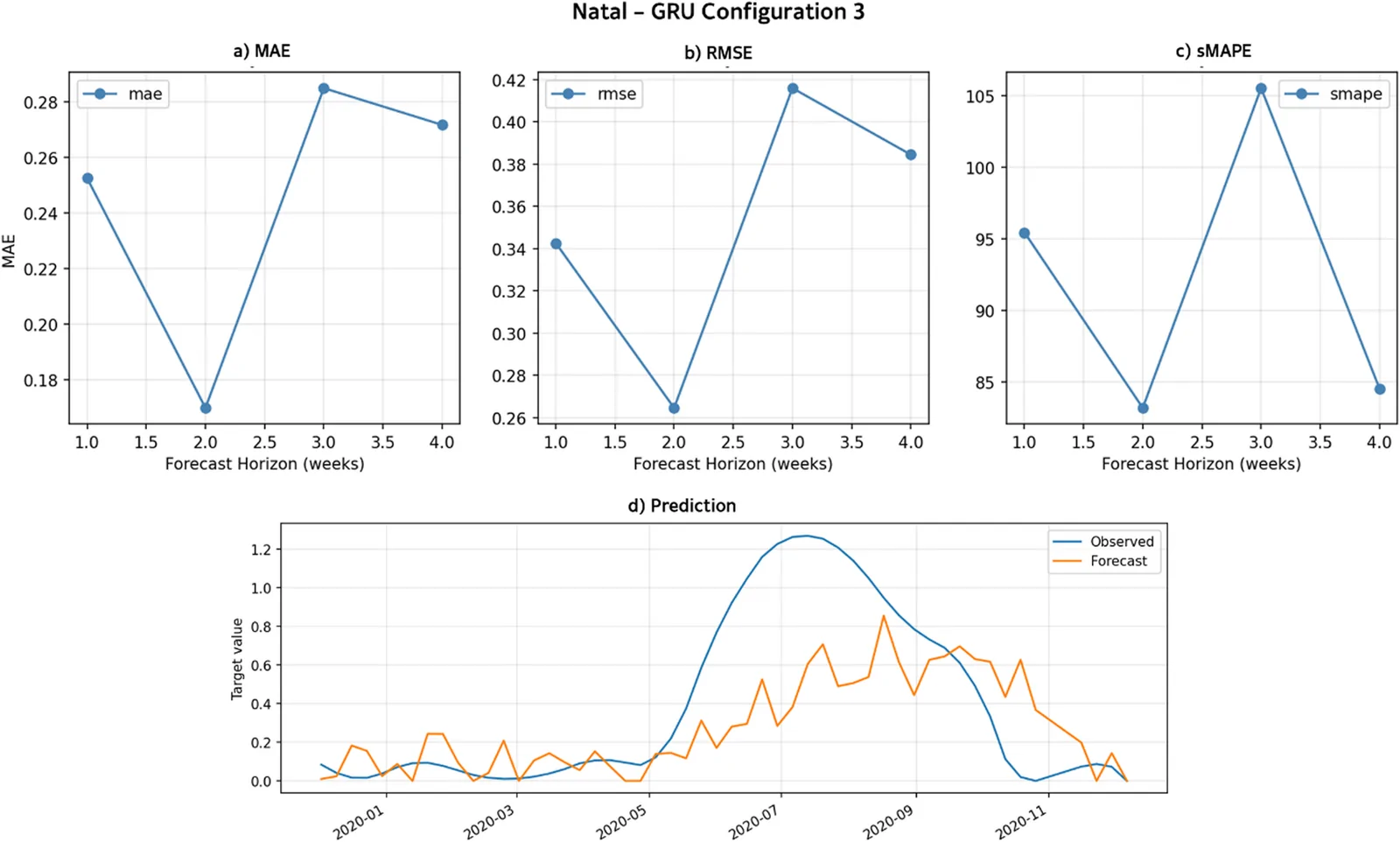
Performance of the GRU model for the capital municipality of Natal

It is noteworthy that the best-performing models, that is, those with the highest positive R² values (Aracaju, São Paulo, Brasília, and Natal), do not present the lowest error values. It is possible to visually analyze the systematic failure of algorithms to capture the magnitude of epidemic peaks in Aracaju (Fig. 2), São Paulo (Fig. 3), Brasília (Fig. 4), and Natal (Fig. 5).

Taieb et al. (2017) discuss the difficulty of algorithms in identifying and operationalizing nonlinear phenomena, due to the complexity of real-world data. Thus, although Aracaju had the highest R² (0.5098) among all capitals, the model failed to predict epidemic outbreaks, demonstrating a delay in the prediction (Fig. 2), which began to grow exponentially around June/July 2020, with a massive peak in 2020. However, as shown in Fig. 2, the model predicts the main outbreak in November/December 2020, when the actual epidemic was already in decline.

For São Paulo (Fig. 3), this delay is also observed, where the model predicts the epidemic peak after the actual outbreak has occurred. On the other hand, as shown in Fig. 4, Brasília showed better temporal alignment, consistently identifying the main period of the outbreak and broadly following the actual peak. However, at the end of the real epidemic peak, the model overestimated the rates. The model for Natal (Fig. 5) also showed a temporal delay and excessive smoothing of the curve, underestimating the severity of the epidemic.

The analysis of errors (MAE, RMSE, sMAPE) across forecast horizons (1 to 4 weeks) reveals an interesting yet problematic pattern. In three of the four cases (Natal, Brasília, and São Paulo), the error does not increase linearly. The error tends to be lower at horizon 2 (H2) and worse at horizons 1, 3, and 4. The sMAPE for São Paulo (Fig. 3), for example, is very high at H1, decreases sharply at H2, and increases again at H3, indicating instability in the model predictions, which may be reasonable for 2 weeks ahead but unstable for 1 or 3 weeks, making them unreliable for operational use.

Therefore, it is observed that a single encoder to predict four distinct horizons resulted in a model unable to capture the volatility and temporal scale of dengue case occurrence, allowing the inference that these are reflections of the limitations of the MIMO method, which has lower flexibility in its predictions (Taieb et al. 2009).

In addition, underfitting is observed, a consequence of the model’s inability to generalize from the data (Aliferis and Simon 2024), demonstrating the GRU’s failure to adequately learn the relationship between the input variables and the output variable, leading to overly simplistic assumptions about the data. The direct consequence is poor performance not only on test data but also on the training data itself (Aliferis and Simon 2024).

The difficulty of the GRU in anticipating peaks, resulting in a clear temporal delay (lag) in its predictions, indicates that the model was not able to capture the complex temporal associations inherent to the system dynamics. This phenomenon may be related to variable delays in biological responses to climatic stimuli, insufficient lag engineering to capture long-term dependencies, or a low signal-to-noise ratio in municipal data, which compromises the network’s predictive capacity in the face of outbreak volatility.

The latter option is most likely, given the stochastic nature of dengue outbreaks, in which the signal-to-noise ratio is reduced, and the biological delays between climatic factors and reported cases are inherently variable. Such factors limit the generalization capacity of the recurrent model, which tends to converge to a conservative solution based on the immediately preceding state of the series. However, determining the exact cause is beyond the scope of this study and presents an opportunity for future investigations involving attention-based architectures or time-series decomposition mechanisms.

It is understood that the causes (e.g., precipitation and temperature) do not produce immediate effects (e.g., dengue outbreaks), contributing to a “natural delay” that may span days or weeks. Therefore, defining a fixed lag is a limitation, suggesting that more advanced methods are required to learn this dynamic directly from the data (Du et al. 2017).

The CatBoost model’s performance, as shown in Table 5, was superior to that of the GRU architectures. The adoption of a Direct forecasting strategy, in which four independent models were trained (one for each horizon H1–H4), allowed the specialist algorithms for each horizon to learn the specific patterns of each week, resulting in higher R² values than those observed for the GRU.

**Table 5:** Performance results of the CatBoost model

Capital municipality	MAE	RMSE	*R*²	sMAPE
Aracaju	0.1461	0.2205	0.6875	68%
Belém	0.0078	0.0105	0.4140	120%
Belo Horizonte	0.0112	0.0182	0.8733	40%
Boa Vista	0.0101	0.0161	0.8317	111%
Brasília	0.3723	0.6165	0.4122	46%
Campo Grande	0.1161	0.1864	0.7582	26%
Cuiabá	0.0151	0.0219	0.8181	64%
Curitiba	0.0058	0.0112	0.4412	122%
Florianópolis	0.3096	0.6264	-0.2385	126%
Fortaleza	0.0674	0.0900	0.8728	36%
Goiânia	0.3997	0.5920	0.9199	31%
João Pessoa	0.0879	0.1171	0.9024	58%
Macapá	0.0072	0.0115	0.6335	123%
Maceió	0.0837	0.1129	0.7807	73%
Manaus	0.0325	0.0418	0.7963	38%
Natal	0.1469	0.2158	0.7715	63%
Palmas	0.2226	0.4030	0.6457	66%
Porto Alegre	0.2219	0.4575	-0.2064	160%
Porto Velho	0.1596	0.2258	0.8643	30%
Recife	0.0191	0.0245	0.5474	25%
Rio Branco	0.0416	0.0638	0.6966	62%
Rio de Janeiro	0.0046	0.0064	0.8282	75%
Salvador	0.0222	0.0293	0.7645	64%
São Luís	0.0624	0.1024	0.8516	33%
São Paulo	0.0075	0.0132	0.8347	60%
Teresina	0.3822	0.6335	0.7907	52%
Vitória	0.0245	0.0524	0.4559	139%

According to Taieb et al. (2009), the Direct strategy decomposes the complex task of forecasting multiple steps ahead, rather than optimizing a single generalist model. In the Direct approach, each model is dedicated exclusively to a specific forecast horizon and optimized for that horizon, which significantly improves the models’ fit to the actual morbidity rate patterns and reduces temporal delay in the predictions.

These results suggest that model choice should consider the structure of the epidemiological series, as also observed in recent health forecasting applications using GRU and gradient boosting models in Brazilian capitals (Nery et al. 2026). GRU-based recurrent models may be more suitable for long and dense time series with stable temporal dependencies, whereas CatBoost may be preferable for sparse, noisy, and peak-driven dengue series represented through engineered lags and moving-window statistics. In such cases, a Direct strategy can improve short-term forecasting by allowing each horizon to be modeled separately (Taieb et al. 2009).

However, as observed for the GRU models, the municipalities with the highest R² values—Goiânia (R² = 0.9199), João Pessoa (R² = 0.9024), Fortaleza (R² = 0.8728), and Belo Horizonte (R² = 0.8733)—did not present the lowest MAE, RMSE, and sMAPE values. Rio de Janeiro, Curitiba, and Macapá showed the lowest MAE and RMSE values, while Recife, Campo Grande, Porto Velho, and Goiânia presented the lowest sMAPE values.

Absolute error metrics, such as MAE and RMSE, are highly influenced by the magnitude of the data. Therefore, capitals with historically low morbidity rates and without large-amplitude outliers naturally exhibited numerically lower absolute errors. It is also noteworthy that, in the case of CatBoost, although these were not the capitals with the highest R² values, those with the lowest errors (MAE and RMSE) also presented satisfactory R² values: Rio de Janeiro: 0.8282; Curitiba: 0.4412; Macapá: 0.6335; and São Paulo: 0.834, in contrast to what was observed for the GRU models.

**Fig. 6:**
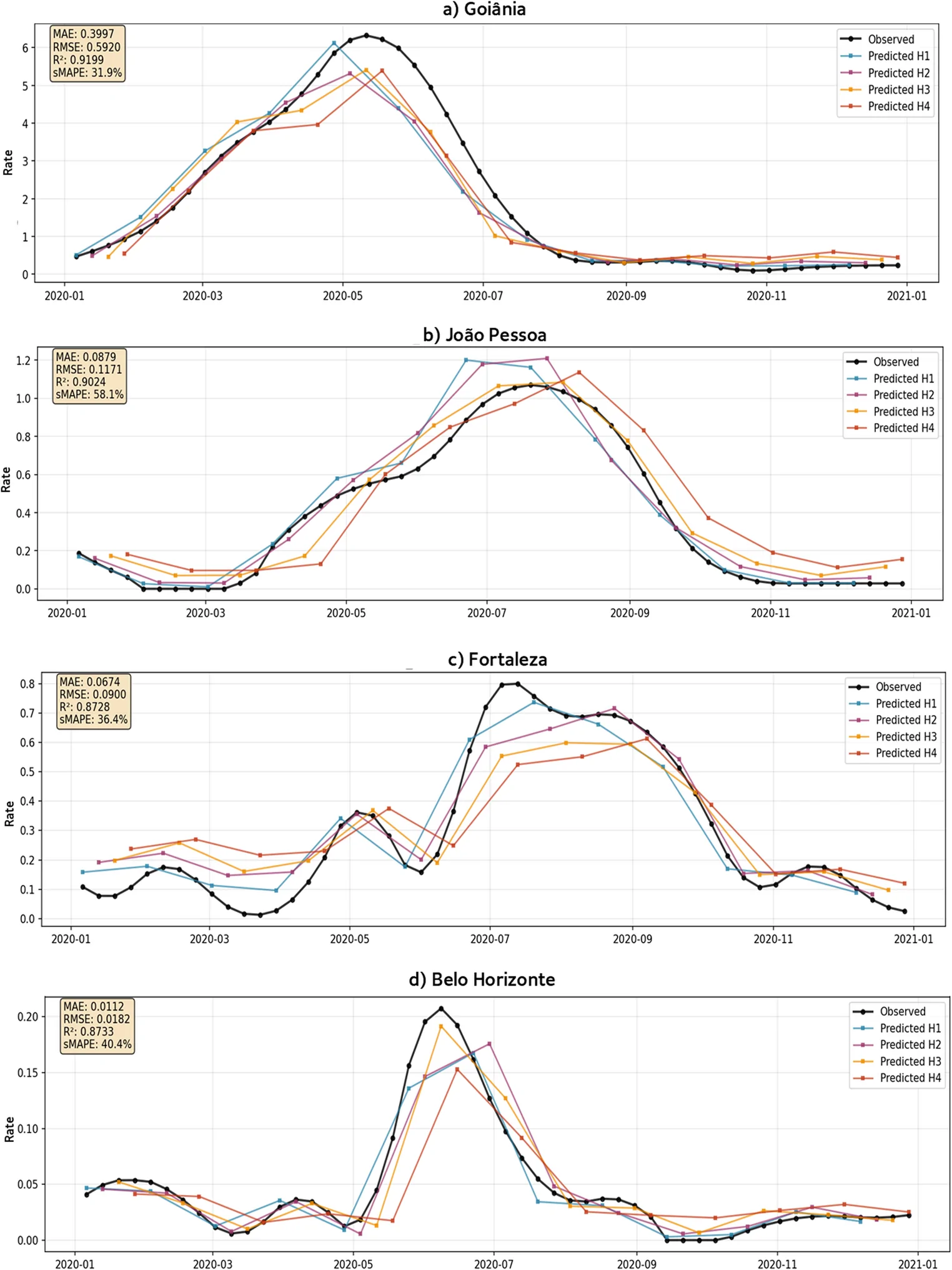
Performance of the predictions using CatBoost for: (**a**) Goiânia; (**b**) João Pessoa; (**c**) Fortaleza; and (**d**) Belo Horizonte, capital municipalities with the highest R² values

Illustrating what was described regarding the error metrics, as can be observed in Fig. 6a, Goiânia presented higher MAE and RMSE values, even with an almost perfect fit (R² > 0.90), successfully capturing the dynamics, temporal scale, and shape of the epidemic curve. In this context, R² emerges as a more robust indicator for comparing the quality of fit across different cities, while MAE and RMSE are more appropriate for understanding the magnitude of operational error within a specific locality.

The performance in João Pessoa (R2 = 0.9024), shown in Fig. 6b, demonstrates the forecast’s adherence to the observed curve. The main inference is that the input features were stable and consistent predictors for this locality, such that the four forecast horizons (H1 to H4) remained very close to each other and to the actual line, indicating that the model maintained its accuracy.

Fortaleza (Fig. 6c) had more epidemic peaks than Goiânia and João Pessoa, making it more challenging for the model to capture these critical moments. However, it successfully understood this dynamic, resulting in an sMAPE of 36.4%. Finally, Fig. 6d shows the prediction for Belo Horizonte, which, despite lower morbidity rates than those of the other municipalities discussed, still achieved a good fit and high model sensitivity. In this case, CatBoost proved capable of predicting larger epidemics and identifying and capturing short-term outbreaks of lower magnitude with greater precision. This suggests that biases did not exclusively drive the model toward high morbidity rates and more severe events, making it an effective tool for surveillance in low-transmission scenarios.

Despite the complexity of the data used, the CatBoost architecture proved efficient, as Gradient Boosting algorithms are considered suitable for handling heterogeneous tabular data, being more effective at selecting truly relevant features and at efficiently handling categorical data (Prokhorenkova et al. 2018).

However, CatBoost failed categorically in the capitals of the Southern Region of Brazil (Florianópolis and Porto Alegre), presenting negative R² values, which may reflect a smaller amount of data, since, according to the data available in the dashboard, the southern region of Brazil is historically characterized by a low incidence of dengue.

In addition, there are reports of the coincidence of dengue epidemic outbreaks during months when temperature conditions were more favorable for transmission (Lee et al. 2021), such that tropical climate zones and those under monsoon influence, as well as humid subtropical climate zones, are more susceptible to the effects of temperature on dengue (Damtew et al. 2023), suggesting the need for further local investigations, since the epidemiological dynamics of dengue may have been influenced by isolated climatic events in the Southern Region of Brazil (Collischonn et al. 2018).

Despite satisfactory results with CatBoost, this study presents methodological and scope limitations that should be addressed in future studies, such as the failure of the GRU-MIMO architecture, which led to systemic underfitting across all capitals, characterized by attenuated predictions and temporal delays.

The second limitation is, coincidentally, the lack of CatBoost model adjustment for the Southern Region, suggesting that the feature engineering based on national-level data was not adequate for local application. Therefore, using a standardized hyperparameter configuration for CatBoost across all 27 capitals does not reflect local model optimization. Therefore, the superior overall performance of CatBoost should not be interpreted as universal model superiority, but as greater suitability to the data structure and forecasting design evaluated in this study.

In practical terms, short-term dengue forecasts may help translate model outputs into anticipatory public health actions. Forecasts up to four weeks ahead can indicate periods and capital municipalities with greater expected risk, supporting the prioritization of epidemiological and entomological surveillance, health-service preparedness, and educational campaigns before transmission intensifies. Therefore, the proposed framework should be understood as a decision-support tool for preventive planning, rather than as a deterministic prediction system or a substitute for established surveillance routines (Sebastianelli et al. 2024; Chen and Moraga 2025; Gurgel-Gonçalves et al. 2024).

The same predictive information may also support the timing and spatial targeting of integrated interventions, including household inspections, removal of breeding sites, larval control, and other vector-control measures. In contexts where dengue vaccination is available, forecasts may complement local epidemiological evidence by helping identify priority periods and territories for vaccination-related planning, always following official eligibility criteria and immunization guidelines. This perspective reinforces that dengue management depends on coordinated surveillance, vector control, community engagement, and vaccination strategies, rather than on isolated interventions (Sanabani 2025; Wilder-Smith et al. 2025).

## Conclusion

This study compared machine learning strategies for short-term forecasting of the weekly dengue morbidity rate in the 27 Brazilian capital municipalities, highlighting the GRU-MIMO architecture with recurrent limitations, such as underfitting, temporal delay, and low capacity to anticipate epidemic peaks, while the CatBoost model with a Direct strategy demonstrated greater robustness in handling the variability and irregularity of epidemiological time series.

The comparison under the same temporal validation framework indicates that greater architectural complexity does not necessarily imply better predictive performance in operational applications. In this context, ensemble-based approaches proved more suitable for supporting short-term epidemiological surveillance in heterogeneous settings such as Brazil. Although limitations related to regional heterogeneity and the representation of local dynamics in models persist, the results contribute to improving predictive systems for public health decision-making.

Thus, the main contribution of this study lies in demonstrating that, for operational dengue surveillance, model selection should prioritize predictive stability and temporal alignment with epidemic dynamics rather than architectural complexity alone. 

## Supplementary Material

Below is the link to the electronic supplementary material.

Supplementary Material 1

## Data Availability

The datasets generated and analyzed during the current study are available from the corresponding author upon reasonable request.

## Abbreviations

CatBoost: Categorical Boosting
DATASUS: Departamento de Informática do Sistema Único de Saúde
GDP: Gross Domestic Product
GRU: Gated Recurrent Unit
IBGE: Instituto Brasileiro de Geografia e Estatística
IPEA: Instituto de Pesquisa Econômica Aplicada
LULC: Land Use and Land Cover
MAE: Mean Absolute Error
MIMO: Multi-Input Multi-Output
PPP: Purchasing Power Parity

## References

[CR1] Aliferis C, Simon G (2024) Overfitting, underfitting and general model overconfidence and under-performance pitfalls and best practices in machine learning and AI. Artificial intelligence and machine learning in health care and medical sciences: Best practices and pitfalls 477–524. 10.1007/978-3-031-39355-6_10 39836821

[CR2] Andrade AC, Falcão LAD, Borges MAZ, Leite ME, Espírito Santo MMD (2024) Are land use and cover changes and socioeconomic factors associated with the occurrence of dengue fever? A case study in Minas Gerais state, Brazil. Resources 13(3):38. 10.3390/resources13030038

[CR3] BRASIL (2024) Ministério da Saúde. Secretaria de Vigilância em Saúde. Monitoramento das arboviroses e balanço de encerramento do Comitê de Operações de Emergência (COE) dengue e outras arboviroses 2024. Boletim Epidemiológico 55(11). https://www.gov.br/saude/pt-br/centrais-de-conteudo/publicacoes/boletins/epidemiologicos/edicoes/2024/boletim-epidemiologico-volume-55-no-11.pdf/view (2024). Accessed 11 November 2025

[CR4] Chen X, Moraga P (2025) Assessing dengue forecasting methods: a comparative study of statistical models and machine learning techniques in Rio de Janeiro, Brazil. Trop Med health 53(1):52. 10.1186/s41182-025-00723-7 40211309 10.1186/s41182-025-00723-7 PMC11984044

[CR5] Cho K, Van Merriënboer B, Gulçehre Ç, Bahdanau D, Bougares F, Schwenk H, Bengio Y (2014) Learning phrase representations using RNN encoder-decoder for statistical machine translation. In Proceedings of the 2014 conference on empirical methods in natural language processing (EMNLP). 1724–1734

[CR6] Codeco C, Coelho F, Cruz O, Oliveira S, Castro T, Bastos L (2018) Infodengue: A nowcasting system for the surveillance of arboviruses in Brazil. Revue d’Épidémiologie. et de Santé Publique 66:S386. 10.1016/j.respe.2018.05.408

[CR7] Collischonn E, Dubreuil V, Mendonça FDA (2018) Relações entre o clima e saúde: o caso da dengue no Rio Grande do Sul no período de 2007 a 2017. Confins revue franco-brésilienne de géographie/Revista franco-brasilera de geografia 37. 10.4000/confins.15431

[CR8] Cordeiro DC, Fonseca FLA, Arab C, Leitão FNC, Zangirolami-Raimundo J, Raimundo RD (2020) Factors associated with dengue cases in brazilian industrial area: an ecological study. J Hum Growth Dev 30(3):451–460. 10.7322/jhgd.v30.11113

[CR9] Damtew YT, Tong M, Varghese BM et al (2023) Effects of high temperatures and heatwaves on dengue fever: a systematic review and meta-analysis. EBioMedicine 91. 10.1016/j.ebiom.2023.104582 10.1016/j.ebiom.2023.104582 PMC10149186 37088034

[CR10] Du S, Song G, Han L, Hong H (2017) Temporal causal inference with time lag. Neural Comput 30(1):271–291. 10.1162/neco_a_01028 29064787 10.1162/neco_a_01028

[CR11] Duarte JL, Diaz-Quijano FA, Batista AC, Giatti LL (2019) Climatic variables associated with dengue incidence in a city of the Western Brazilian Amazon region. Rev Soc Bras Med Trop 52:e20180429. 10.1590/0037-8682-0429-2018 30810657 10.1590/0037-8682-0429-2018

[CR12] Guo P, Liu T, Zhang Q et al (2017) Developing a dengue forecast model using machine learning: A case study in China. PLoS Negl Trop Dis 11(10):e0005973. 10.1371/journal.pntd.0005973 29036169 10.1371/journal.pntd.0005973 PMC5658193

[CR13] Gurgel-Gonçalves R, Oliveira WKD, Croda J (2024) The greatest dengue epidemic in Brazil: surveillance, prevention, and control. Rev Soc Bras Med Trop 57:e00203–2024. 10.1590/0037-8682-0113-2024 39319953 10.1590/0037-8682-0113-2024 PMC11415067

[CR14] IBGE (2022) Instituto brasileiro de geografia e estatística. https://www.ibge.gov.br/pt/inicio.html. Accessed 26 July 2024

[CR15] Ibrahim AA, Ridwan RL, Muhammed MM, Abdulaziz RO, Saheed GA (2020) Comparison of the CatBoost classifier with other machine learning methods. Int J Adv Comput Sci Appl 11(11):738–748. 10.14569/IJACSA.2020.0111190

[CR16] Johansson MA, Apfeldorf KM, Dobson S et al (2019) An open challenge to advance probabilistic forecasting for dengue epidemics. Proc Natl Acad Sci 116(48):24268–24274. 10.1073/pnas.1909865116 31712420 10.1073/pnas.1909865116 PMC6883829

[CR17] Kikuti M, Cunha GM, Paploski IA et al (2015) Spatial distribution of dengue in a Brazilian urban slum setting: role of socioeconomic gradient in disease risk. PLoS Negl Trop Dis 9(7):e0003937. 10.1371/journal.pntd.0003937 26196686 10.1371/journal.pntd.0003937 PMC4510880

[CR18] Kokonendji CC, Bonat WH, Abid R (2021) Tweedie regression models and its geometric sums for (semi-) continuous data. Wiley Interdisciplinary Reviews: Comput Stat 13(1):e1496. 10.1002/wics.1496

[CR19] Kurz CF (2017) Tweedie distributions for fitting semicontinuous health care utilization cost data. BMC Med Res Methodol 17(1):171. 10.1186/s12874-017-0445-y 29258428 10.1186/s12874-017-0445-y PMC5735804

[CR20] Lee SA, Economou T, Barcellos C, Catão R, Carvalho MS, Lowe R (2021) Effect of climate change, connectivity, and socioeconomic factors on the expansion of the dengue virus transmission zone in 21st century Brazil: an ecological modelling study. Lancet Planet Health 5:S14. 10.1016/S2542-5196(21)00098-X

[CR21] Lim B, Arık SÖ, Loeff N, Pfister T (2021) Temporal fusion transformers for interpretable multi-horizon time series forecasting. Int J Forecast 37(4):1748–1764. 10.1016/j.ijforecast.2021.03.012

[CR22] Lowe R, Barcellos C, Coelho CA et al (2014) Dengue outlook for the World Cup in Brazil: an early warning model framework driven by real-time seasonal climate forecasts. Lancet Infect Dis 14(7):619–626. 10.1016/S1473-3099(14)70781-9 24841859 10.1016/S1473-3099(14)70781-9

[CR23] Mussumeci E, Coelho FC (2020) Large-scale multivariate forecasting models for Dengue-LSTM versus random forest regression. Spat spatio-temporal Epidemiol 35:100372. 10.1016/j.sste.2020.100372 10.1016/j.sste.2020.100372 33138951

[CR24] Nery LM, Nicomedes NP, Camargo PCMG, Outeiro SA, Lusquino Filho LAD, Farias CM, Silva DCC (2026) Analysis of models to estimate morbidity rates of respiratory diseases through deep learning. Tropical Med Int Health 31(6):742–753. 10.1111/tmi.70126 10.1111/tmi.70126 PMC13246585 41856921

[CR25] Nicomedes NP, Camargo PCMG, Arenas LAO, Nery LM, Lusquino-Filho LAD, Silva DCC (2026) Spatial patterns of mortality from respiratory diseases and their relationship with socio-environmental indicators in Brazil. Bioscience J 42(e42012):1981–3163. 10.14393/BJ-v42n0a2026-78449

[CR26] PAHO WHO (2024) Pan American health organization; World health organization epidemiological update: Increase in dengue cases in the Region of the Americas – 18 June 2024. Washington, D.C.: Pan American health organization / world health organization. https://www.paho.org/sites/default/files/2024-06/2024-june-18-phe-epi-update-dengue-en2.pdf. Accessed 11 November 2025

[CR27] Prokhorenkova L, Gusev G, Vorobev A, Dorogush AV, Gulin A (2018) CatBoost: unbiased boosting with categorical features. Advances in neural information processing systems 31

[CR28] Rissanen M, Inklaar R (2023) A comparison of different sources of purchasing power parity (PPPs) estimates. World Bank Group. https://shorturl.at/6jZ3g Accessed 04 July 2024

[CR29] Sanabani SS (2025) Epidemiology of dengue in Brazil: recent trends and public health response. Discover Public Health 22(1):518. 10.1186/s12982-025-00937-4

[CR30] Sebastianelli A, Spiller D, Carmo R et al (2024) A reproducible ensemble machine learning approach to forecast dengue outbreaks. Sci Rep 14(1):3807. 10.1038/s41598-024-52796-9 38360915 10.1038/s41598-024-52796-9 PMC10869339

[CR31] Simon LM, Rangel TF (2021) Are temperature suitability and socioeconomic factors reliable predictors of dengue transmission in Brazil? Front Trop Dis 2:758393. 10.3389/fitd.2021.758393

[CR32] Sobral MFF, Sobral AIGP, (2019) Casos de dengue e coleta de lixo urbano: um estudo na Cidade do Recife, Brasil. Ciência & Saúde Coletiva 24:1075-1082. 10.1590/1413-81232018243.10702017 10.1590/1413-81232018243.10702017 30892527

[CR33] Sousa SC, Carneiro M, Eiras ÁE, Bezerra JMT, Barbosa DS (2021) Factors associated with the occurrence of dengue epidemics in Brazil: a systematic review. Revista Panam de Salud Pública 45:e84. 10.26633/RPSP.2021.84 10.26633/RPSP.2021.84 PMC8344382 34377143

[CR34] Souza C, Maia P, Stolerman LM, Rolla V, Velho L (2022) Predicting dengue outbreaks in Brazil with manifold learning on climate data. Expert Syst Appl 192:116324. 10.1016/j.eswa.2021.116324

[CR36] Taieb SB, Bontempi G, Sorjamaa A, Lendasse A (2009) Long-term prediction of time series by combining direct and mimo strategies. In 2009 International Joint Conference on Neural Networks. 3054–3061. 10.1109/IJCNN.2009.5178802

[CR37] Taieb SB, Sorjamaa A, Bontempi G (2010) Multiple-output modeling for multi-step-ahead time series forecasting. Neurocomputing 73(10–12):1950–1957. 10.1016/j.neucom.2009.11.030

[CR35] Taieb A, Soltani M, Chaari A (2017) Parameter optimization of MIMO fuzzy optimal model predictive control by APSO. Complexity 2017(1):5813192. 10.1155/2017/5813192

[CR38] Viana DV, Ignotti E (2013) A ocorrência da dengue e variações meteorológicas no Brasil: revisão sistemática. Revista brasileira de epidemiologia 16:240–256. 10.1590/S1415-790X2013000200002 24141998 10.1590/S1415-790X2013000200002

[CR39] WHO (2024) World Health Organization. Dengue – global situation. Disease outbreak news, Geneva. https://www.who.int/emergencies/disease-outbreak-news/item/2024-DON518. Accessed 11 November 2025

[CR40] WHO (2025) World Health Organization. Dengue and severe dengue: fact sheet. Geneva: World Health Organization. https://www.who.int/news-room/fact-sheets/detail/dengue-and-severe-dengue. Accessed 11 November 2025

[CR41] Wilder-Smith A, Cherian T, Hombach J (2025) Dengue vaccine development and deployment into routine immunization. Vaccines 13(5):483. 10.3390/vaccines13050483 40432095 10.3390/vaccines13050483 PMC12115503

[CR42] Xavier AC, Scanlon BR, King CW, Alves AI (2022) New improved Brazilian daily weather gridded data (1961–2020). Int J Climatol 42(16):8390–8404. 10.1002/joc.7731

[CR43] Xu H, Lou Y (2023) Multivariate time series anomaly detection: a hybrid method based on GRU-SAE and gain. In 2023 IEEE 6th information technology, networking, electronic and automation control conference (ITNEC) 6:32–38

[CR44] Zellweger RM, Cano J, Mangeas M et al (2017) Socioeconomic and environmental determinants of dengue transmission in an urban setting: An ecological study in Nouméa, New Caledonia. PLoS Negl Trop Dis 11(4):e0005471. 10.1371/journal.pntd.0005471 28369149 10.1371/journal.pntd.0005471 PMC5395238

