# Supplementary material for: Comparative evaluation of machine learning strategies for short-term dengue forecasting in Brazilian capital municipalities
Source: Int J Biometeorol. 2026 Aug 14;70(8):233. doi: 10.1007/s00484-026-03300-7 (PMC13476183; doi:10.1007/s00484-026-03300-7)
Supplement: Supplementary file 1 [file 484_2026_3300_MOESM1_ESM.docx]

**Supplementary Table 1**. Descriptive summary of the dengue morbidity rate in the 27 Brazilian capital municipalities, 1999–2021.

| **Year** | **Mean** | **Median** | **SD** | **CV (%)** | **IQR (Q1–Q3)** | **Minimum** | **Maximum** | **Zero (%)** |
| --- | --- | --- | --- | --- | --- | --- | --- | --- |
| 1999 | 0,1751 | 0 | 0,3474 | 198% | 0,0000–0,1712 | 0 | 1,8535 | 59% |
| 2000 | 0,3419 | 0 | 0,8308 | 243% | 0,0000–0,2200 | 0 | 5,3880 | 55% |
| 2001 | 0,6167 | 0,1472 | 1,2271 | 199% | 0,0000–0,6199 | 0 | 9,0550 | 38% |
| 2002 | 1,1727 | 0,3395 | 2,0521 | 175% | 0,0574–1,2743 | 0 | 12,3088 | 22% |
| 2003 | 1,2535 | 0,3684 | 2,5296 | 202% | 0,0149–1,2660 | 0 | 21,5579 | 24% |
| 2004 | 0,5072 | 0,1727 | 1,0253 | 202% | 0,0000–0,5329 | 0 | 10,8361 | 32% |
| 2005 | 1,0035 | 0,2671 | 2,0023 | 200% | 0,0183–0,9575 | 0 | 11,9746 | 23% |
| 2006 | 1,0996 | 0,3154 | 2,7391 | 249% | 0,0417–0,9332 | 0 | 24,2541 | 23% |
| 2007 | 2,4238 | 0,6881 | 5,3712 | 222% | 0,1382–2,2423 | 0 | 59,6254 | 17% |
| 2008 | 3,5683 | 0,6309 | 7,8895 | 221% | 0,0782–2,6700 | 0 | 60,5526 | 19% |
| 2009 | 2,5289 | 0,6127 | 5,6037 | 222% | 0,1534–1,8801 | 0 | 39,5484 | 16% |
| 2010 | 4,0216 | 1,7717 | 6,8047 | 169% | 0,5046–3,9405 | 0 | 44,3174 | 10% |
| 2011 | 2,8205 | 1,3508 | 4,5591 | 162% | 0,4344–3,0078 | 0 | 42,7303 | 10% |
| 2012 | 1,6330 | 0,8262 | 2,7166 | 166% | 0,2296–2,0366 | 0 | 21,3778 | 12% |
| 2013 | 1,2444 | 0,3989 | 2,7592 | 222% | 0,1620–1,1632 | 0 | 25,1096 | 14% |
| 2014 | 0,8036 | 0,4091 | 1,4033 | 175% | 0,1186–0,8755 | 0 | 12,8862 | 17% |
| 2015 | 1,5291 | 0,4740 | 4,6490 | 304% | 0,1594–1,0520 | 0 | 42,8462 | 12% |
| 2016 | 1,1792 | 0,2652 | 3,1633 | 268% | 0,0672–0,8351 | 0 | 29,7201 | 20% |
| 2017 | 0,5098 | 0,0955 | 1,5791 | 310% | 0,0000–0,3389 | 0 | 16,5063 | 33% |
| 2018 | 0,5383 | 0,1541 | 1,5122 | 281% | 0,0000–0,4131 | 0 | 13,7059 | 30% |
| 2019 | 1,8123 | 0,3974 | 4,0178 | 222% | 0,0651–1,6447 | 0 | 35,2697 | 21% |
| 2020 | 0,6394 | 0,1618 | 1,6398 | 256% | 0,0000–0,4853 | 0 | 15,7821 | 32% |
| 2021 | 0,6259 | 0,1208 | 1,4121 | 226% | 0,0000–0,5465 | 0 | 12,2069 | 35% |

IQR: Interquartile range; SD: Standard deviation; CV: Coefficient of variation
